# Quantitative Analysis of Cytokinesis *In Situ* during *C. elegans* Postembryonic Development

**DOI:** 10.1371/journal.pone.0110689

**Published:** 2014-10-20

**Authors:** Karine G. Bourdages, Benjamin Lacroix, Jonas F. Dorn, Carlos P. Descovich, Amy S. Maddox

**Affiliations:** 1 Institute for Research in Immunology and Cancer, Université de Montréal, Montréal, Québec, Canada; 2 Advanced Quantitative Sciences, Novartis Pharma AG, Basel, Switzerland; 3 Department of Biology, University of North Carolina, Chapel Hill, North Carolina, United States of America; University of Toledo, United States of America

## Abstract

The physical separation of a cell into two daughter cells during cytokinesis requires cell-intrinsic shape changes driven by a contractile ring. However, *in vivo*, cells interact with their environment, which includes other cells. How cytokinesis occurs in tissues is not well understood. Here, we studied cytokinesis in an intact animal during tissue biogenesis. We used high-resolution microscopy and quantitative analysis to study the three rounds of division of the *C. elegans* vulval precursor cells (VPCs). The VPCs are cut in half longitudinally with each division. Contractile ring breadth, but not the speed of ring closure, scales with cell length. Furrowing speed instead scales with division plane dimensions, and scaling is consistent between the VPCs and *C. elegans* blastomeres. We compared our VPC cytokinesis kinetics data with measurements from the *C. elegans* zygote and HeLa and *Drosophila* S2 cells. Both the speed dynamics and asymmetry of ring closure are qualitatively conserved among cell types. Unlike in the *C. elegans* zygote but similar to other epithelial cells, Anillin is required for proper ring closure speed but not asymmetry in the VPCs. We present evidence that tissue organization impacts the dynamics of cytokinesis by comparing our results on the VPCs with the cells of the somatic gonad. In sum, this work establishes somatic lineages in post-embryonic *C. elegans* development as cell biological models for the study of cytokinesis *in situ*.

## Introduction

Cytokinesis is the last step of cell division, physically partitioning the cytoplasm of a cell into two daughter cells. Cytokinesis failure results in tetraploidy, which promotes p53 activation and in most cases cell cycle arrest [1], but in several conditions, further proliferation [2], [3]. Due to their supernumerary centrioles, dividing tetraploid cells exhibit errors in spindle bipolarity and chromosome segregation [4], [5]. The resulting aneuploidy implicates cytokinesis failure in oncogenic transformation [4]. Interestingly, regulated cytokinesis failure occurs during differentiation of several cell types including hepatocytes and cardiomyocytes (reviewed in [6]).

To initiate cytokinesis, the geometry of the anaphase spindle dictates the local activation of the small GTPase Rho. Active, GTP-bound RhoA activates formin actin nucleators and non-muscle myosin II, and recruits other effectors including the scaffolding protein Anillin (reviewed in [7]–[10]). Filament sliding and/or depolymerization are thought to drive closure of the actomyosin contractile ring and membrane furrowing [8], [11], [12]. While it is well accepted that spindle signaling converges on Rho to elicit actomyosin ring assembly and closure, reports of cell-type specific requirements for spindle and contractile ring components [13]–[16] suggest that distinct mechanisms can achieve the common goal of cell division.

Recent comparative studies have yielded novel insights into the general principles of cytokinesis mechanics. One unifying concept is that actomyosin rings are built from discrete “contractile units” [17], [18]. This model was posed to explain how ring closure speed scales with cell size [17]. Furrow speed scaling is observed in diverse cell types [17], [19], indicating that this phenomenon occurs as a result of a conserved feature of actomyosin rings.

Our current understanding of cytokinesis stems mainly from using isolated cells including yeasts, mammalian cultured cells and invertebrate zygotes, such as that of *Caenorhabditis elegans*. However, it is not well known how the widely accepted the molecular dynamics and mechanics of cytokinesis applies to cells in the context of living tissues. Defining mechanistic differences in cytokinesis among cell types may help explain the tissue specificity of gene requirements during development and of drug sensitivity in some cancers. Here, we examined the impact of tissue context on cytokinesis as it occurred in the simple epithelium of a living animal.

Epithelia are ubiquitous tissues that regulate homeostasis and act as barriers against the surrounding environment (reviewed in [20]). Epithelial cells are polarized, with their apical domain facing the lumen or outside environment and the basolateral surface contacting neighboring cells and the basement membrane. Cadherin-based adherens junctions delineate these domains and mechanically and biochemically connect epithelial cells [21]. When epithelial cells divide such that both daughter cells inherit the apical domain, their intercellular junctions must be remodeled. How epithelial integrity is preserved throughout this process is not fully understood.

Recent work with the *Drosophila* embryo, pupal notum and follicular epithelia has provided insights into the requirements for cytokinesis *in vivo* [22]–[25]. These complementary studies described how intercellular adhesions mechanically oppose forces in the contractile ring, causing it to close non-concentrically. Thus, the geometry of contractile ring closure is not completely cell-autonomous and accordingly, ring asymmetry does not require Anillin and septins as it does in the *C. elegans* zygote [22], [23], [25], [26]. Interestingly, epithelial cells in these various tissues appear to differently regulate junction remodeling during division [27], [28]. Adhesions in the division plane become disengaged on both sides of the dividing cell in the embryo, on only one side in follicle cells, and not at all in the notum [27], [28]. The differences in how tissue context impacts cytokinesis among tissues may relate to their specialized functions.

Here, we characterize cytokinesis *in situ* using *C. elegans*, studying somatic cell divisions during post-embryonic development. We took advantage of the simplicity and thorough cell fate characterization of the egg laying apparatus in *C. elegans*, specifically, the vulval precursor cells (VPCs). VPC size reduced by half with each round of division, and the dimensions of the contractile ring scaled with cell size. Quantitative analysis of the kinetics of cytokinesis in the VPCs revealed acceleration and deceleration of the ring, which we also observed in diverse cell types including human HeLa and *Drosophila* S2 cultured cells. As in other epithelial cells, furrowing in the VPCs was asymmetric, terminating towards the apical domain. Examining furrowing in HeLa and S2 cultured cells revealed that asymmetry also occurs in these “isolated” cells, and is polarized towards the substrate. Thus, asymmetry can arise from mechanical resistance originating from various cellular features. While the scaffolding protein Anillin was not required for the asymmetry of VPC furrowing, its depletion slowed cytokinesis in these cells. Depletion of conserved intercellular adhesion components did not significantly alter the kinetics or geometry of VPC cytokinesis, suggesting that junctions are exceptionally robust in this tissue. In cells of the less organized somatic gonad, furrowing was more symmetric and slower than in the VPCs. Collectively, this work contributes to establishing tissues of the developing *C. elegans* as cell biological systems for studying cell division.

## Results

### Visualization of the vulval precursor cells (VPCs) *in situ*

**Figure 1:**
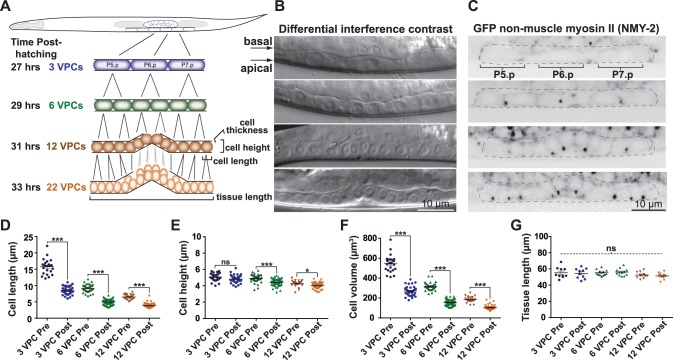
The *C. elegans* vulval precursor cells (VPCs) inside the living and developing animal. (A) Schematic representation of the VPCs (P5.p, P6.p and P7.p cells) in a third larval stage (L3) worm. 3 VPC stage: undivided precursors (purple); daughter cells: 6 VPC stage (green); 12 granddaughter cells: 12 VPC stage (orange); final 22 descendants: 22 VPC stage. (B) DIC images of the VPCs at the corresponding stages shown in A. For all images, anterior is to the left and dorsal is to the top. Scale bar = 10 µm. (C) Maximum intensity projection images of worms expressing GFP-tagged non-muscle myosin II (NMY-2) in the VPCs (dotted lines) at the 3, 6, 12 and 22 VPC stages. Scale bar = 10 µm (D–E) Scatter plots of individual VPC measurements before (pre) and after (post) each round of VPC division. Cell length: ***: p<0.0001, unpaired t-test. Cell height: n.s.: p = 0.06, ***: p<0.0001, *: p = 0.02, unpaired t-test. Bars = mean with SEM. n(cells) >20 and n(worms) ≥8 for each VPC stage. (F) VPC volume = length (D)×height (E) x thickness (number of 0.6 µm steps occupied by the cells). ***: p<0.0001, unpaired t-test. Bars = mean with SEM. n(cells) >20 and n(worms) ≥8 and for each VPC stage. (G) Length of region occupied by the VPCs and their descendants. n.s.: p>0.1, one-way analysis of variance. Bars = mean with SEM. n(worms) ≥10 for each stage.

To study cytokinesis *in situ*, we sought a simple, well-characterized epithelium. The nematode *C. elegans* lays eggs via the vulva, which starts as a simple epithelium, comprising the vulval precursor cells (VPCs; Figure 1A). The VPCs’ lineage and placement are invariant, and the morphogenetic events of vulva formation are well understood [29]–[33]. At the third larval stage of *C. elegans* development, six cells (P3.p–P8.p) are competent to form the vulva [30], [32], [34]. Upon induction, only three of these cells, P5.p, P6.p and P7.p, adopt vulval fate [30], [32], [34], [35]. Over the course of 6 hours at 25°C, they undergo three rounds of division to generate 22 descendants, which further go through morphogenesis to form the vulva [36], [37] (Figure 1A).

To visualize VPC divisions in living animals, we performed high-resolution microscopy of a worm strain expressing GFP-tagged non-muscle myosin II (NMY-2; hereafter, “myosin”), a core component of the contractile ring, under the control of its own promoter. This transgenic strain has been widely used and is considered a faithful reporter of active myosin [17], [38]–[40]. At the beginning of the third larval stage (L3), which we denote as the 3 VPC stage, the P5.p, P6.p and P7.p cells’ basal surfaces are internal and their apical domains lay against the worm’s ventral cuticle (Figure 1A–C). In interphase, myosin was present at the cortex and enriched at apical junctions between VPCs that also likely contain cytokinetic midbody remnants (Figure 1C). In the mid-L3 stage the three VPCs divided in the plane of the epithelium, giving rise to 6 daughter cells (the 6 VPC stage; Figure 1A–C). Approximately two hours later they underwent a second round of division to produce 12 granddaughter cells. During the early 12 VPC stage prior to the last round of division and the L3/L4 molt, descendants of the P6.p cell invaginated dorsally by apical constriction (Figure 1A–C). 10 of 12 granddaughter cells undergo a third and final round of division, giving rise to 22 descendants (Figure 1A–C). Thus, high-resolution imaging of a fluorescent *C. elegans* strain allowed us to observe the organization of the VPCs during early vulva development.

To characterize the VPCs as a cell biological model, we first determined the dimensions of the VPCs (see Figure 1A). VPC length was reduced by approximately half from one round of division to the next, while the height (apical-basal cell axis) and thickness (left-right worm axis) of VPCs remained more constant (Figure 1D and E). Thus, cell volume was reduced by approximately half during each of the three rounds of VPC divisions (Figure 1F). Consistent with the decrease in VPC length, the three rounds of division occurred without appreciable growth of the tissue (Figure 1G). Thus, VPC divisions are reductional within the epithelium, providing an opportunity to study the effects of cell size on various aspects of cell division.

### Contractile ring dimensions scale with cell size

**Figure 2:**
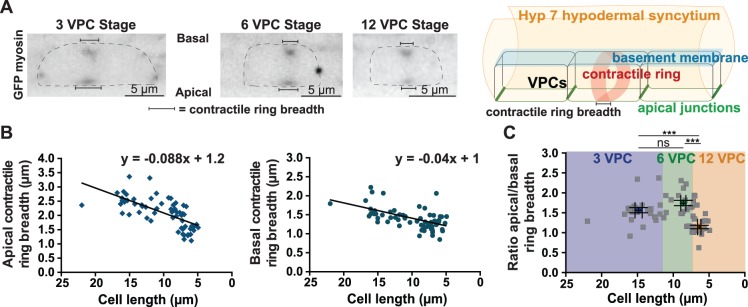
Contractile ring dimensions scale with the length of VPCs. (A) GFP-tagged myosin worms at the 3 (left), 6 (middle) and 12 (right) VPC stages. Images are maximum intensity projections taken at <150 seconds following cytokinesis onset. Myosin is enriched in the contractile ring at both the basal (upper) and apical (lower) domain of the cells. Dotted lines: dividing cells. Scale bar = 5 µm. Right: 3D schematic of a dividing VPC showing contractile ring breadth in brackets. (B) Apical (left) and basal (right) contractile ring breadth plotted against VPC length for all three rounds of division. The x-axis was inverted to show the decrease in cell length through divisions. Best-fit linear regressions and their equations are shown. (C) Scatter plot of apical versus basal contractile ring breadth (data from B) for cell lengths at the 3 (purple), 6 (green) and 12 (orange) VPC stages. Colored dots: average for each stage. Scale bars = mean with SEM for both axes. n.s.: p value = 0.05, ***: p<0.0001, unpaired t-test.

We took advantage of the progressive reduction in VPC size to test how different aspects of cytokinesis scale with cell size. We first tested if the contractile ring scales with cell size, as has been demonstrated for meiotic and mitotic spindles [41]–[43]. We used worms expressing GFP-tagged myosin to visualize the contractile ring at each of the three rounds of VPC division. At cytokinesis onset, myosin was enriched in an equatorial band encircling the cell and visible on both the apical and basal domain of the dividing cell (Figure 2A). We measured the breadth of the contractile ring (how much of the cell’s long axis was occupied by myosin) along both the cell’s apical and basal surfaces and compared it to cell length. As the VPCs become smaller from one round of division to the next, so do the apical and basal dimensions of the contractile ring (Figure 2B). This is consistent with the idea that contractile ring dimensions relate to spindle size, which scales with cell length. Contractile rings were broader at the apical region than the basal domain of the cell (Figure 2C), indicating that apicobasal cell polarity generates differences in the mechanical or biochemical mechanisms that focus the contractile ring. The extent of this difference varied among the three rounds of VPC divisions; the apical region of the contractile ring was significantly broader than at the basal surface during the first two rounds of VPC division, but nearly identical during the last round of cytokinesis (Figure 2C). This result indicates that there is a lower bound to contractile ring breadth.

### Furrowing speed scales with division plane dimensions

**Figure 3:**
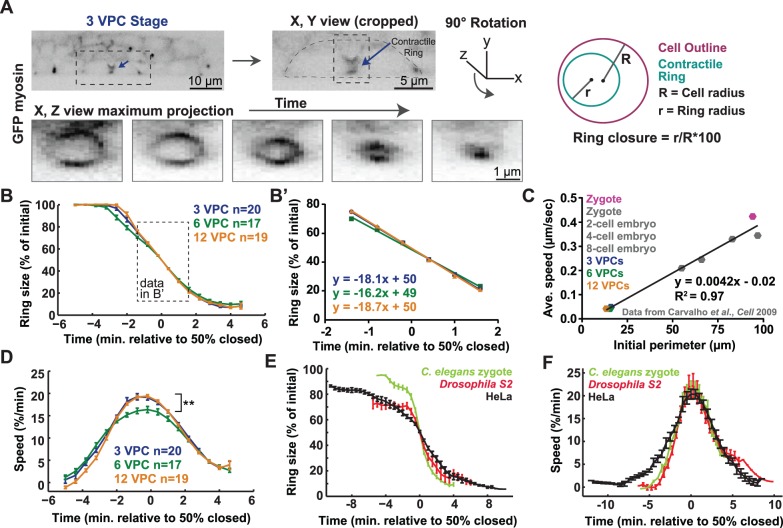
Quantitative analysis of the kinetics of contractile ring closure in the VPCs. (A) Maximum intensity projection images of a worm expressing myosin::GFP at the 3 VPC stage. Left: dotted box: dividing cell; arrow: contractile ring. Scale bar = 10 µm. Middle: enlargement of the dividing cell (dotted outline); dotted box and arrow: contractile ring. Scale bar = 5 µm. The cropped contractile ring is rotated 90° to generate a z, x maximum projection. Bottom: contractile ring closure over time. Scale bar = 1 µm. Right: representation of ring annotation and the parameters quantified: red = cell outline; green = contractile ring; R = cell radius; r = ring radius; Ring closure = r/R*100. (B) Average percentage of contractile ring closure over time aligned at the midpoint of closure for the first (purple), second (green) and third (orange) rounds of VPC cytokinesis. Purple/first: n(cells) = 20, n(worms) = 11. Green/second: n(cells) = 17, n(worms) = 6. Orange/third: n(cells) = 19, n(worms) = 8. Error bars = SEM. Dotted box: data for B’. (B’) Linear regression lines and their equations for 20%–80% ring closure (data from B). (C) Furrowing speed versus division plane perimeter (3 VPC: purple, 6 VPC: green and 12 VPC: orange, grey: data from [17]; pink: our zygote measurement). Linear regression fitted to all 8 data points. (D) Average speed of contractile ring closure over time for the three rounds of VPC cytokinesis. First round/purple, n(cells) = 20, n(worms) = 11, second/green, n(cells) = 17, n(worms) = 6, third/orange, n(cells) = 19, n(worms) = 8. **: p = 0.006 for 6 versus 12 VPC stage at time 0, unpaired t-test. Error bars = SEM. (E–F) Graphs of average percentage of ring closure and speed over time for HeLa cells (black), *Drosophila* S2 cells (red) and the *C. elegans* zygote (light green). *C. elegans* zygote: n(cells) = 9, *Drosophila* S2: n(cells) = 5, HeLa: n(cells) = 8. Error bars = SEM.

Another aspect of contractile ring biology that scales with cell size is furrowing speed, such that larger cells furrow more quickly than smaller cells of a given cell type [12], [17], [19], [44]. One possibility was that speed scales with total available myosin or other contractile ring components and thus cell volume. According to this model, since VPCs halve their volume at each round of cell division (Figure 1F), furrow speed would decrease with decreasing VPC size. Alternatively, Calvert and colleagues presented evidence that furrowing speed scales with division plane dimensions [19]. VPC height and thickness, and therefore division plane circumference, remain roughly constant (Figure 1E and F), so furrowing speed would be expected to be similar among the three rounds of division. We performed time-lapse imaging through the thickness of the VPCs and measured contractile ring closure (Figure 3A and B). Indeed, furrowing speed, represented by the average speed between 20% and 80% ring closure, is relatively similar among rounds of VPC division, and does not scale with VPC volume (Figure 3B’). Maximum furrowing speed is significantly lower in the middle round of VPC divisions, but also does not scale with VPC size (see Figure 3D).

We next explored whether the scalability of furrowing speed with cell size extends outside of a given cell type. We compared our furrowing speed data from the VPCs (Figure 3B’) with those measured previously in *C. elegans* blastomeres, where furrowing speed scales with cell size in general [17]. Strikingly, our measurements from VPCs fit very well with the correlation between division plane diameter and furrowing speed in the much larger blastomeres (Figure 3C). Thus, scaling is a conserved phenomenon whose arithmetic relationship holds among different cell types in *C. elegans*. Our results indicate that furrow speed is dictated by a feature of the contractile ring that is universal among cells of varying sizes, shapes, fates and tissue contexts.

### Contractile ring closure occurs via acceleration and deceleration

Measuring VPC contractile ring closure with high temporal resolution, we noticed that its speed is not constant but rather accelerated until reaching a maximum speed of approximately 18% per minute near 50% closure, and then decelerated until closure (Figure 3D). To examine whether contractile rings in well-studied model cell types also accelerate and decelerate, we measured ring closure over time in the *C. elegans* zygote, HeLa human cultured cells, and *Drosophila* S2 cells (Figure 3E). We then calculated how the speed of closure changed with time (Figure 3F). In all these cell types, contractile ring closure accelerated until the ring was approximately half closed, and then decelerated (Figure 3F). Thus, gradual change in ring closure speed appears to be a general characteristic of metazoan cytokinesis.

### Asymmetric furrowing occurs towards the apical membrane of VPCs

**Figure 4:**
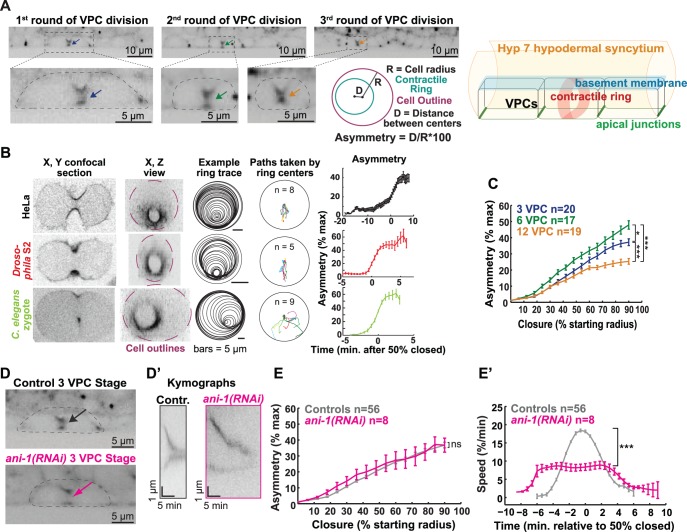
Strong intercellular adhesion leads to robust asymmetric contractile ring closure in the VPCs. (A) First (left), second (middle) and third (right) rounds of VPC cytokinesis in myosin::GFP worms. First round dividing cell reproduced from Figure 3A for ease of comparison. Scale bar = 10 µm. Dotted boxes and outlines: dividing cells; arrows: contractile rings. Scale bar = 5 µm. Representation of ring annotation and the parameters quantified: red = cell outline; green = contractile ring; R = cell radius; D = distance between cell and ring centers; Asymmetry = D/R*100. 3D schematic of contractile ring closure in the VPCs. (B) First column: x, y view of dividing HeLa cell (top), *Drosophila* S2 cell (middle), and *C. elegans* zygote (bottom). Second column: corresponding x, z views. Third column: example contractile ring location over time. Scale bar = 5 µm. Fourth column: the path taken by the ring for all examples of each cell type. Last column: asymmetry versus time. (C) Average asymmetry of furrowing over the percentage of VPC ring closure (first round; purple, second; green and third; orange). First: n(cells) = 20, n(worms) = 11. Second: n(cells) = 17, n(worms) = 6. Third: n(cells) = 19, n(worms) = 8. Error bars = SEM. ***: p<0.0001, *: p = 0.03, unpaired t-test calculated at 80% closed. (D) Confocal images of control and ANI-1 depleted worms expressing myosin::GFP (3 VPC stage). Dotted lines: dividing cells; arrows: ingressing furrows. Scale bar = 5 µm. (D’) Kymographs of contractile ring closure for control (black) and ANI-1 depleted (fuchsia) worms expressing myosin::GFP. Vertical scale bar = 1 µm. Horizontal scale bar = 5 min. (E-E’) Average furrow asymmetry and ring closure speed (all three rounds of VPC cytokinesis). Control (grey), n(cells) = 56, n(worms) = 25. *ani-1(RNAi)* (fuchsia), n(cells) = 8, n(worms) = 4. Error bars = SEM. n.s.: p>0.1, unpaired t-test. ***: p<0.0001, unpaired t-test.

Since contractile ring dimensions and closure speed scaled with VPC length and division plane dimensions, respectively, we next explored whether another feature of cytokinesis scaled with VPC size. Asymmetric cytokinesis (also called polarized, non-concentric, or unilateral cytokinesis) was first characterized in the *C. elegans* zygote, where confinement in the eggshell suggests it occurs cell-autonomously [26], [45]. Asymmetric cytokinesis has been observed in many epithelial cells and neuroepithelial cells *in situ* [22]–[25], [46]–[49]. Recently, it was demonstrated with the *Drosophila* embryonic blastoderm and follicular epithelia that asymmetric furrowing in epithelial cells can be explained by apical junctions resisting the inward pulling forces of the contractile ring [23], [25]. Strikingly, ring closure is asymmetric and invariantly polarized to the substrate in human HeLa and *Drosophila* S2 cells (Figure 4B). These results suggest that remnant substrate adhesions can also resist furrow forces and direct polarized ring closure. Thus, furrow asymmetry appears to be universal among metazoan cell types, but can occur by multiple mechanical means.

Contractile ring closure was asymmetric in the VPCs (Figure 4A and C; Movie S1). Ingression was polarized towards the apical membrane (Figure 4A; Movie S1), as in other epithelia [22]–[25], [47]–[49]. The extent of asymmetry differed among the three rounds of VPC division (Figure 4C). Asymmetry increased from the first to the second round of division, but then decreased for the third round, where it was the most symmetric (Figure 4C). Thus, asymmetry did not scale with VPC dimensions.

The polarity of asymmetric furrowing suggested that the intercellular contiguity of apical junctions resists the inward pulling forces of the contractile ring, as was demonstrated with the *Drosophila* embryonic blastoderm and follicular epithelia [23], [25]. Unfortunately, technical difficulties prohibited us from drawing conclusions on the roles of intercellular adhesions in the geometry of VPC cytokinesis (Figure S1; see Discussion).

In the *C. elegans* zygote, ANI-1^Anillin^ is required for asymmetric contractile ring closure [26]. Targeting of ANI-1 during post-embryonic development led to gross defects in vulval morphogenesis and vulval protrusion (Pvl), as previously seen [50]. Depletion of ANI-1 from the VPCs did not alter furrow asymmetry (Figure 4D and E), suggesting that furrow asymmetry is not ring-intrinsic but is caused by mechanical resistance by the apical junctions. Similar results and conclusions were obtained with *Drosophila* epithelial cells *in situ* [22], [23], [25]. ANI-1 depletion from VPCs slowed contractile ring closure (Figure 4D’ and E’), as in mammalian cultured cells injected with an Anillin antibody [51], and in the *Drosophila* embryonic and notum epithelial cells depleted of Anillin [22], [23]. Interestingly, this effect of ANI-1 depletion on furrowing speed is not seen in the *C. elegans* zygote [26]. Thus, ANI-1^Anillin^ is differentially required for cytokinesis in epithelial cells versus early blastomeres in *C. elegans*.

### Epithelial organization influences the kinetics of cytokinesis

**Figure 5:**
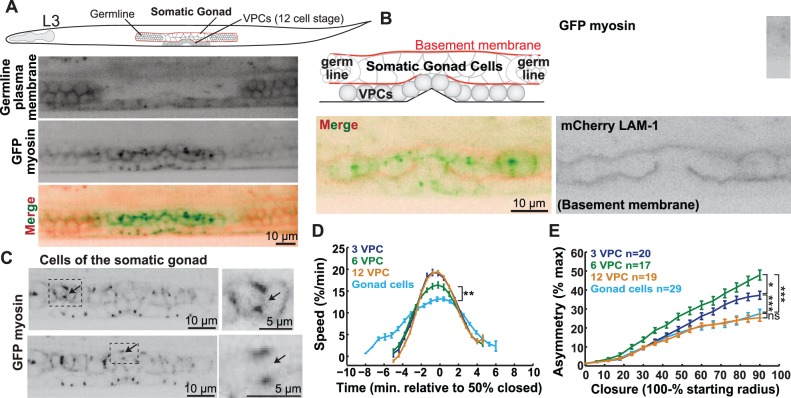
Tissue geometry influences the kinetics of cytokinesis in the cells of the somatic gonad. (A) Schematic representing an L3 worm (∼31 hours post-hatching), showing the somatic gonad, VPCs and germline. Worm expressing mCherry-tagged phospholipase C (PLCδ) PH domain in the germline (top), GFP-tagged myosin in the VPCs and somatic gonad (middle), and merge image (bottom). Scale bar = 10 µm. (B) Schematic of the cells of the somatic gonad (middle) and the germline (extremities) surrounded by a continuous basement membrane (red). Worm at the 12 VPC stage expressing myosin::GFP and mCherry::LAM-1 (laminin-1) to mark basement membrane. Scale bar = 10 µm. (C) Maximum intensity projection images of the somatic gonad expressing GFP myosin. Dotted boxes: dividing cells, enlarged to the right; arrows: contractile ring. Scale bars = 10 µm (left); 5 µm (right). (D-E) Average ring closure speed and asymmetry (somatic gonad: teal blue, first VPC division: purple, second: green, and third: orange). Each VPC stage n(cells) ≥17, n(worms) ≥6. Gonad cells; n(cells) = 29, n(worms) = 12. Error bars = SEM. **: p = 0.0023, unpaired t-test, gonad cells versus 6 VPC stage. *: p = 0.06, ***: p<0.0001, n.s.: p>0.1, unpaired t-test.

The epithelium containing the VPCs is highly organized: the single layer of cells are all of similar size, with apparently similar contact with the basement membrane and neighboring epithelial cells [52] (Figure 1). In contrast, the *C. elegans* somatic gonad, located interior to the VPCs, is an ovoid collection of cells surrounded by a basement membrane (Figure 5A and B). The somatic gonad cells, which are segregated towards the worm midline from the two arms of the developing germline (Figure 5A), undergo several rounds of division to give rise to the cells that encase the germline, spermatheca and uterus [53], [54]. To test how epithelial organization influences cytokinesis, we measured the kinetics and geometry of cytokinesis in the somatic gonad cells and compared them to our results with the VPCs.

The contractile rings of the somatic gonad cells could be observed via time-lapse imaging of GFP-tagged myosin, as for VPCs (Figure 5C; Movie S2). Contractile ring closure was slower in the somatic gonad cells than in the slowest VPCs (at the second round of division; Figure 5D). Contractile ring closure was also more concentric, resembling that of the third round of VPC division (Figure 5E). Thus, as in the VPCs, furrowing speed is not strictly correlated with asymmetry, indicating that they are influenced by independent aspects of the contractile ring. These results suggest that tissue organization influences the dynamics of contractile ring closure.

## Discussion

Here, we set out to establish a system for studying cytokinesis *in situ*. We used high-resolution microscopy of a strain expressing GFP-tagged myosin to follow contractile ring closure over the three rounds of vulval precursor cell (VPC) cytokinesis within developing *C. elegans*. We also examined a distinct tissue, the somatic gonad. By characterizing cytokinesis in multiple settings and taking a four dimensional view of the contractile ring, we defined conserved features of cytokinesis, lending insight into general principles of contractile ring function.

We first established that the VPCs reduced in length by approximately half upon each division (Figure 1). This cell size reduction allowed us to investigate scalability of the contractile apparatus. The width of the contractile ring scaled with VPC length (Figure 2A and B), supporting the hypothesis that the dimensions of the mitotic spindle dictate the site of contractile ring assembly [39], and parallels the finding that spindle length scales to cell size [42], [43], thus adding to a growing body of knowledge on organelle scaling.

When we investigated whether other aspects of cytokinesis scaled with cell size, we found that the speed of furrowing scaled with division plane dimensions and not with overall cell size. Whether furrowing speed scales with division plane size or cell volume had not been discernable from the study of *C. elegans* blastomeres [17], but had been arrived at using filamentous fungus [19]. Interestingly, the scaling of furrowing speed with the size of the division plane is consistent among the VPCs and blastomeres of *C. elegans* (Figure 3C). Carvalho and colleagues suggested that this scaling occurs because rings are constructed from standard sized contractile units, and large rings contain more contractile units than smaller cells [17]. Our results thus suggest that different cell types of a given species possess that same contractile unit.

In measuring how the speed of contractile ring closure changes over time, we noticed that it first increases and then decreases (Figure 3D). Although this phenomenon is not widely appreciated, it has been reported [18], [55]–[58]. Careful inspection of data from cells throughout phylogeny reveals that when ring size is plotted over time, the resulting curve is sigmoidal (first concave downward and later concave upward), due to acceleration and deceleration [19], [26], [59]. Acceleration may reflect progressive contractile ring compaction and organization, while deceleration may result from limitations on contractile ring disassembly. Understanding the structural bases of acceleration and deceleration and the switch between these two states will no doubt lead to insights into general principles of cytokinesis.

As observed in diverse epithelial cells [22]–[25], [47]–[49], VPC contractile rings close in a polarized, apically-directed fashion (Figure 4A; Movie S1). In *Drosophila* epithelia, the polarity of furrowing and resulting apical positioning of the midbody promote the formation of a long interface between daughter cells, important for epithelial integrity in a proliferating tissue [24], [25]. Thus, asymmetry results in this specific advantage for epithelial cells. However, we also observed asymmetry in non-epithelial cell types (Figure 4B). Together with several elegant mechanical perturbations of epithelial cytokinesis [22]–[25], our data suggest that asymmetry is an inevitable result of mechanical resistance in one region of the division plane.

Previous work in ectodermal and follicular epithelial cells of *Drosophila* established that mechanical resistance by apical adherens junctions dictates the polarity of asymmetric contractile ring ingression [23], [25]. Although we assume that the same principle holds for the *C. elegans* VPCs, we did not observe more concentric closure of the contractile ring upon depletion of either HMR-1^E-cadherin^ or AJM-1, principal components of the two adhesion subdomains (Figure S1A–D’). Simultaneous RNAi for these two targets did not exacerbate the effects on vulval morphogenesis and were not examined at the cell level (Figure S1E). These results suggest that VPC apical junctional integrity is robust due to redundant and/or persistent intercellular adhesion proteins. Cadherin- and AJM-1-based adhesion complexes are redundant during embryonic morphogenesis of the *C. elegans* gut epithelium [60]. In addition, junction proteins may have persisted despite RNAi, since the vulval epithelium is relatively insensitive to RNAi [61]. Our attempts to circumvent this issue using a mutant strain (*rrf-3* pk1426) with increased RNAi sensitivity [62] did not enhance the penetrance of terminal vulval defects (Figure S1F) and thus were not pursued further. It is possible that the VPCs’ junctions with the Hyp7 hypodermis (orange in Figure 2A), which lie in the division plane for longitudinal VPC divisions, are generally more compliant and/or less depleted by RNAi. Lastly, the VPCs’ apical association with the cuticle could contribute to mechanical resistance by the apical aspect of these cells. Indeed, the cuticle-associated apical ECM was implicated in the maintenance of apical junction integrity in the *C. elegans* excretory system [63].

In sum, our characterization of cytokinesis in the VPCs of *C. elegans* lays the foundation for applying the wealth of knowledge that exists on vulval genetics and morphogenesis to the study of cytokinesis. It also provides insights into the differences in mechanisms and geometry of cell division *in situ* versus in isolated cells. These distinctions could aid the understanding and development of cancer therapies, since one of the major challenges of anti-mitotic agents is their unexplained tissue specificity [64].

## Materials and Methods

### C. *elegans* strains

The following strains were used:
JJ1473 *(zuIs45 [nmy-2::NMY-2::GFP+unc-119(+)] V),*
FT250 *(xnIs96 [pJN455(hmr-1p::HMR-1::GFP::unc-54 3′UTR)+unc-119(+)]),*
SU93 *(jcIs1 [ajm-1::GFP+unc-29(+)+rol-6(su1006)] IV),*
OD183: OD70 *(ltIs44 [pAA173, pie-1p::mCherry::PH(PLC1delta1)+unc-119(+)])*×JJ1473, and JJ1473×NK574 *(qyIs86 [cog-2::GFP]; lam-1::cherry)*.

### C. *elegans* culture

*C. elegans* strains were maintained at 25°C using standard procedures [65]. For live imaging and feeding experiments worms were synchronized at the first larval stage (L1) using alkaline bleach (1.2% NaOCl, 250 mM KOH) [66]. Control L3 worms were mounted for imaging at 27 hours post-hatching. Somatic gonad imaging was performed using late L3 worms between 31 to 33 hours post-hatching (12 VPC stage).

### RNA-mediated interference

Protein depletions were carried out by placing 10 to 15 worms on a plate seeded with the HT115 bacterial strain containing the L4440 vector inducing IPTG mediated dsRNA expression, as described [67]. Single bacterial clones from the Ahringer library [68], [69], kindly provided by Jean-Claude Labbé (IRIC, Université de Montréal), were sequenced to confirm the presence of target genes. Synchronized L1 worms were fed dsRNA expressing bacteria for >26 hours at 25°C before imaging. To assess the effects of the RNAi on overall vulval morphogenesis, worms were grown for >72 hours (to adulthood) and scored for protruded vulva (Pvl) phenotypes using a stereomicroscope.

### Worm mounting and imaging

Worms anesthetized in 0.01% tetramisole in M9 buffer for 10 minutes were mounted on a 5% agarose pad bearing 20 to 80 µm wide grooves made by a custom nanofabricated silica plate. Worms were overlaid with a poly-L-Lysine coated coverslip. To prevent desiccation, tetramisole solution was added between the coverslip and agarose pad and the chamber was sealed with VaLaP (1∶1∶1 Vaseline, lanolin and paraffin). Imaging was performed using a Swept Field Confocal (SFC, Nikon Canada, Mississauga, ON, Canada; and Prairie Technologies, Madison, WI, USA). The 50 µm slit mode was used with or without 2×2 binning on a CoolSnap HQ2 camera (Photometrics, Tucson, AZ). We used a 60X/1.4 NA Plan-Apochromat objective, 0.6 µm z steps, 30 seconds intervals, and either 400 or 600 milliseconds exposures. All acquisition settings, including laser intensity, were controlled using NIS-Elements software (Nikon). Time-lapse imaging of VPCs and somatic gonad cells was performed for several hours to capture multiple divisions (each lasting approximately 10 minutes). Only one acquisition was made per worm. n≥3 worms per condition.

### Cell and contractile ring dimension measurements

VPC and tissue dimensions were measured with NIS-Elements software (Nikon). The length and height of VPCs were measured before and after division. Cell thickness was estimated by counting the number of 0.6 µm z steps occupied by the cell. Cell volume was calculated by multiplying length, height and thickness. Tissue length was determined by a longitudinal measure of the three VPCs and their descendants before and after each round of division. Measurements were recorded in Excel (Microsoft) and graphed using Prism (GraphPad software). Statistical analyses for cell height (unpaired t-test) and tissue length (one-way ANOVA) were performed in Prism. For cell length and volume, unpaired t-tests were performed in MATLAB. Contractile ring dimensions in the VPCs were assessed with a custom MATLAB-based software. Original acquisitions were processed (cell cropping) for analysis in NIS-Elements (Nikon). Contractile ring dimensions from maximum intensity projection images were measured in the plane of imaging (x, y view). Apical and basal contractile ring breadths were measures of the equatorial region enriched for myosin. Myosin enrichment was defined by higher shades of grey compared with adjacent myosin at the cell perimeter. Breadth measurements for the first five time points with detectable equatorial myosin were averaged. Measurements were compiled in Excel; statistical analysis and graphing were performed using Prism.

### Fluorescence intensity measurements

To assess the extent of HMR-1 and AJM-1 depletions, the fluorescence intensity of GFP-tagged HMR-1 and AJM-1 was measured using FT250 and SU93 strains, respectively. All imaging parameters were held constant between control and RNAi animals. All measurements of fluorescence intensity were performed in the same way using Fiji (ImageJ 1.48a, NIH). Images were opened as 16-bit.nd2 files. A single confocal slice was selected for analysis. Images were rotated to align the VPCs horizontally. A box was drawn over the entire region of the VPCs. Each measurement was normalized to the fluorescence intensity of the background outside the worm. Mean fluorescence intensity was recorded in Excel. Graphing and statistical analysis were performed in Prism.

### Cytokinesis kinetics analysis

Quantification of the kinetics of cytokinesis was performed using custom MATLAB software cyanRing (CYtokinesis ANalysis of the contractile RING) [70]. Individual cells were first cropped from time-lapse z series in x and y using NIS-Elements. Using cyanRing, the contractile ring was cropped and rotated to generate a maximum intensity projection image of the division plane (z, x view). The outline of the cell and contractile ring were annotated over time by marking three points (Figure 3A). Ring size and position were calculated from best-fit circles through these points. Graphs of ring closure timing, speed and asymmetry were made using cyanRing.

### Statistical analysis

P values for cell length and cell volume measurements were obtained in MATLAB by performing unpaired t-tests. Statistical analyses of cyanRing data were performed in MATLAB using a custom script. Unpaired t-tests were calculated for each specified sets of data. For cell height (unpaired t-test) and tissue length (one-way analysis of variance) measurements, statistical analyses were performed in Prism (GraphPad software). Statistical analyses for fluorescence intensity quantification were also performed in Prism, obtaining p values following unpaired t-tests.

## Supporting Information

Figure S1 **Targeting of intercellular junction proteins by RNAi quantitatively affects some aspects of cytokinesis.** (A) Schematic representation of HMR-1^E-cadherin^ apical localization at the 6 VPC stage in an x, y view. Corresponding images of control and *hmr-1(RNAi)* worms. Scale bar = 10 µm. (A’) HMR-1::GFP intensity in the entire VPC region (6 VPC stage). Green: average intensity for controls; purple: HMR-1 depleted worms. Bars = mean with SEM. ***: p<0.0001, unpaired t-test. (B–B’) Average asymmetry and speed of contractile ring closure, respectively, in controls (green) and HMR-1 depleted worms (purple) during the second round of division. Controls n(cells) = 17, n(worms) = 6. HMR-1 depletions n(cells) = 12, n(worms) = 5. Error bars = SEM. n.s.: p>0.1, unpaired t-test. (C) Schematic of a ventral view of VPCs expressing GFP-tagged AJM-1. Corresponding maximum intensity projection images of 6 VPC stage AJM-1::GFP control and AJM-1 depleted worms. Scale bar = 10 µm. (C’) AJM-1::GFP intensity in control (green) and AJM-1 depleted (light purple) worms (3 and 6 VPC stages). Bars = mean with SEM. n.s.: p = 0.16, unpaired t-test. (D–D’) Average asymmetry and speed of contractile ring closure for controls and AJM-1 depleted worms during the second round of division. Controls (green) n(cells) = 17, n(worms) = 6. *ajm-1(RNAi)* (light purple) n(cells) = 7, n(worms) = 3. Error bars = SEM. n.s.: p>0.1. **: p = 0.0026, unpaired t-test. (E) The penetrance of the protruded vulva (Pvl) phenotype was scored at least 72 hours post-feeding. *hmr-1(RNAi)*: purple, *ajm-1(RNAi)*: light purple, *hmr-1+ajm-1(RNAi)*: striped column. n(replicates): *hmr-1* = 6, *ajm-1* = 5, *hmr-1/ajm-1* = 2. Bars = mean with SEM. (F) Penetrance of the Pvl phenotype for three different worm strains; JJ1473 (NMY-2::GFP), FT250 (HMR-1::GFP) and *rrf-3* (pk1426). The average percentage of Pvl is represented for *ani-1* (fuchsia), *hmr-1* (purple) and *ajm-1* (light purple) depleted worms. n(replicates): JJ1473/*ani-1* n = 5, JJ1473/*hmr-1* n = 6, JJ1473/*ajm-1* n = 5, FT250/*ani-1* n = 1, FT250/*hmr-1* n = 3, FT250/*ajm-1* n = 2 and *rrf-3*/*ani-1*, *rrf-3/hmr-1*, *rrf-3/ajm-1* n = 1.
(EPS)

Movie S1 **Asymmetric furrowing to the apical membrane of VPCs.** Time-lapse movie of a worm expressing GFP-tagged myosin during the second round of VPC division. Anterior is to the left and the apical membrane at the bottom. The contractile ring closes towards the apical membrane of all 6 daughter cells. Images were acquired every 2 minutes at 100x magnification, using a swept field confocal microscope (Nikon). A maximum intensity projection of 0.6 µm z slices is shown. The movie is played at 5 frames/s. Scale bar = 10 µm. Time in minutes.
(MOV)

Movie S2 **Cytokinesis in cells of the somatic gonad.** Time-lapse imaging of a worm expressing GFP-tagged myosin, approximately 31 hours post-hatching. Same worm as shown in Figure 5C. Anterior is to the left and the ventral cuticle at the bottom. Above the 12 VPCs, four cells of the somatic gonad undergo cytokinesis, observed by myosin enrichment in the contractile ring. Images were captured every 30 seconds, at a magnification of 60x, using a swept field confocal microscope (Nikon). A maximum intensity projection of 0.6 µm z slices is shown. The movie is played at 5 frames/s. Scale bar = 10 µm. Time in minutes.
(MOV)
