# Correction: Quantitative Analysis of Cytokinesis *In Situ* during *C. elegans* Postembryonic Development

**DOI:** 10.1371/journal.pone.0116240

**Published:** 2014-12-17

**Authors:** The PLOS ONE Staff

Figure 5B is missing the panel for GFP myosin. The authors have provided a corrected version of Figure 5 here.

**Figure 5:**
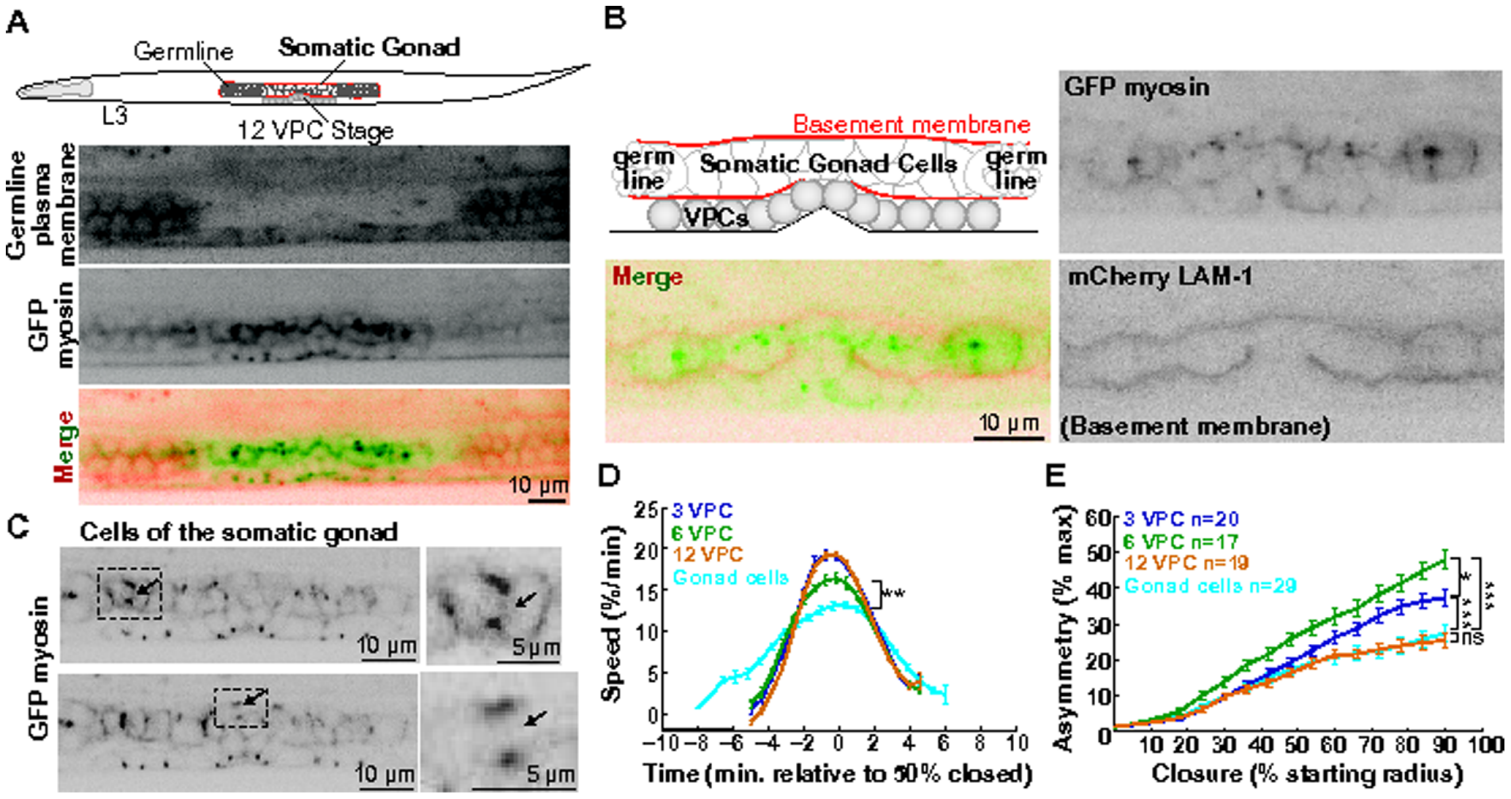
Tissue geometry influences the kinetics of cytokinesis in the cells of the somatic gonad. (A) Schematic representing an L3 worm (∼31 hours post-hatching), showing the somatic gonad, VPCs and germline. Worm expressing mCherry-tagged phospholipase C (PLCδ) PH domain in the germline (top), GFP-tagged myosin in the VPCs and somatic gonad (middle), and merge image (bottom). Scale bar  =  10 µm. (B) Schematic of the cells of the somatic gonad (middle) and the germline (extremities) surrounded by a continuous basement membrane (red). Worm at the 12 VPC stage expressing myosin::GFP and mCherry::LAM-1 (laminin-1) to mark basement membrane. Scale bar  =  10 µm. (C) Maximum intensity projection images of the somatic gonad expressing GFP myosin. Dotted boxes: dividing cells, enlarged to the right; arrows: contractile ring. Scale bars  =  10 µm (left); 5 µm (right). (D-E) Average ring closure speed and asymmetry (somatic gonad: teal blue, first VPC division: purple, second: green, and third: orange). Each VPC stage n(cells) ≥17, n(worms) ≥6. Gonad cells; n(cells)  =  29, n(worms)  =  12. Error bars  =  SEM. **: p  =  0.0023, unpaired t-test, gonad cells versus 6 VPC stage. *: p  =  0.06, ***: p<0.0001, n.s.: p>0.1, unpaired t-test.
